# An Eco-Evolutionary Model for Demographic and Phenological Responses in Migratory Birds

**DOI:** 10.3390/biology1030639

**Published:** 2012-11-14

**Authors:** Jacob Johansson, Isabel M. Smallegange, Niclas Jonzén

**Affiliations:** 1 Department of Biology, Theoretical Population Ecology and Evolution Group, Ecology Bldg, Lund University, SE-22362 Lund, Sweden; Email: niclas.jonzen@biol.lu.se; 2 Division of Biology, Imperial College London, Silwood Park, Ascot, SL5 7PY, UK; Email: i.smallegange@imperial.ac.uk

**Keywords:** phenology, territory competition, climate change, life history, timing, evolutionary game theory, demography, pre-breeding survival

## Abstract

Many migratory birds have changed their timing of arrival at breeding grounds in response to recent climate change. Understanding the adaptive value and the demographic consequences of these shifts are key challenges. To address these questions we extend previous models of phenological adaptation to climate change under territory competition to include feedback from population dynamics, winter survival and habitat productivity. We study effects of improved pre-breeding survival and of earlier food abundance peak. We show that phenological responses depend strongly on equilibrium population density via effects on territory competition. When density is high, improved pre-breeding survival affects selection pressures more than shifts of the resource peak. Under certain conditions, an advanced food peak can even select for later arrival due to competitive release. Improved pre-breeding survival has positive effects on population density that in many cases is stronger than negative effects of an advanced food peak. The fraction of young in the population decreases in all scenarios of change, but food peak shifts only affect population structure marginally unless population density is low. This work thus provides several missing links between phenological adaptation and demographic responses, and augments the toolbox for interpreting ongoing phenological shifts in migratory birds. We illustrate the utility of our model by explaining different patterns in demographic trends and phenological shifts in populations of Pied flycatchers (*Ficedula hypoleuca*) across Western Europe.

## 1. Introduction

Changes in the timing of arrival of long distance migratory birds at their breeding grounds have been observed in many studies. There is a general pattern of advanced arrival among birds that have their breeding grounds in the Northern hemisphere, and these shifts have in turn been connected to increased temperatures due to climate change [1,2,3,4]. Phenological changes in migratory birds have also been related to population trends [5,6,7]. An interesting pattern that has emerged from these observations is that species that show no or only weak phenological change, expressed either as a change in mean/median arrival date [6] or degree-days [8], have decreased in population size. This has in turn been interpreted as an effect of increased temporal mismatch with food resources [8]. Thus, after arrival at the breeding grounds, birds have either little time to complete the necessary steps involved in reproduction, or the offspring’s food demand does not overlap with the peak in availability of high quality food. Reproductive success can be compromised for both these reasons.

Reproductive mismatch is however only one of many ways in which environmental change can affect populations of migratory birds, and it has been argued that the whole life cycle of an organism needs to be taken into account when predicting demographic responses to climate change in general [9,10,11]. The NAO index, which correlates with winter survival, was for example found to be a more important factor to explain population declines than breeding success in a Pied flycatcher population breeding in Britain [12]. Focusing on a single life-cycle event, such as breeding, in isolation from other events can also be misleading because climate change can affect temperatures unevenly over the season. Studies on Pied flycatchers in The Netherlands have indicated that, although temperature changes late in the season affected the timing of the resource peak, temperatures earlier in the season did not change [13]. When changes in different seasonal components are decoupled in this way, negative effects on population trends due to reproductive mismatch may either be reinforced or counteracted by changes in other environmental components affecting e.g., adult survival.

Environmental changes can also have evolutionary consequences and studies have indicated that selective pressures on the phenology of migratory birds have changed [5,14]. An outstanding question in this context is to what extent species can adapt their phenologies to new climatic conditions and thereby avoid negative demographic consequences. Establishing whether an observed phenological shift is adaptive or not, is however a challenging task. Different approaches to deal with this problem have been proposed. As a first approximation, it has been suggested that the temporal shift in food abundance can be used as a yardstick to evaluate the adaptive value of an observed phenological shift of a consumer species [15]. The annual routine approach to modelling phenological adaptation in migratory birds [16,17,18] emphasizes that the optimal phenology depends on the interplay between environmental and physiological state variables over the whole annual cycle. Studies based on evolutionary game theory in turn show that competition for territories can play a crucial role for bird phenology [19]. Interestingly, further theoretical work indicates that the degree of competition can affect whether migratory birds adapt their timing of arrival at the breeding grounds according to shifts in temporal food abundance distributions or to changes in pre-breeding conditions [20,21]. Competition for territories can also influence demographic responses in populations undergoing phenological adaptation. It has for example been shown that in situations where strong competition has selected for early arrival, advanced food resource peaks may well give rise to a temporary increase in population density in migratory birds [22].

In sum, we expect changes in competitive pressures, in conditions for pre-breeding survival, as well as in food resource peaks to have consequences for demographic changes and adaptive phenological responses. The aim of this study is to find out how these factors interact and what their relative roles are. We include these factors in a single framework by combining elements from previous models. The resulting model thus contains pre-breeding survival similar to the implementation by Jonzén *et al*. [20], and a population dynamics module borrowed from a study by Johansson and Jonzén [22]. To study territory competition we furthermore use the model in Johansson and Jonzén [21], where this aspect is treated in a more explicit and mechanistic way compared to the other two studies [20,22]. We also include winter survival and resource abundance as parameters into the models since they can affect competitive pressures indirectly by affecting population densities. Acknowledging the fact that climate change may have uneven effects on different components of the seasonal environment we study scenarios of change where conditions for pre-breeding survival and food resource peak date may change independently of each other.

We use the following procedure to study effects of environmental changes on the system [22]. First we calculate the evolutionarily stable strategy (ESS) for timing of arrival at breeding grounds, and the corresponding demographic equilibrium. Then we simulate climate change by adjusting the model parameters that represent the onset of benign pre-breeding conditions and the timing of the food abundance peak. Finally we study how these changes affect selection pressures, population densities and age structure. The inclusion of population dynamics into the model allows us to study population dynamic feedback on evolution, an aspect that to large extent has been lacking in previous studies. By considering more ecological factors, this new approach enables closer connections between eco-evolutionary theory and data. We conclude the paper by discussing some empirical findings in the light of our results and promising areas for future research.

## 2. Model and Methods

### 2.1. Model for Population Dynamics and Phenological Adaptation

**Figure 1:**
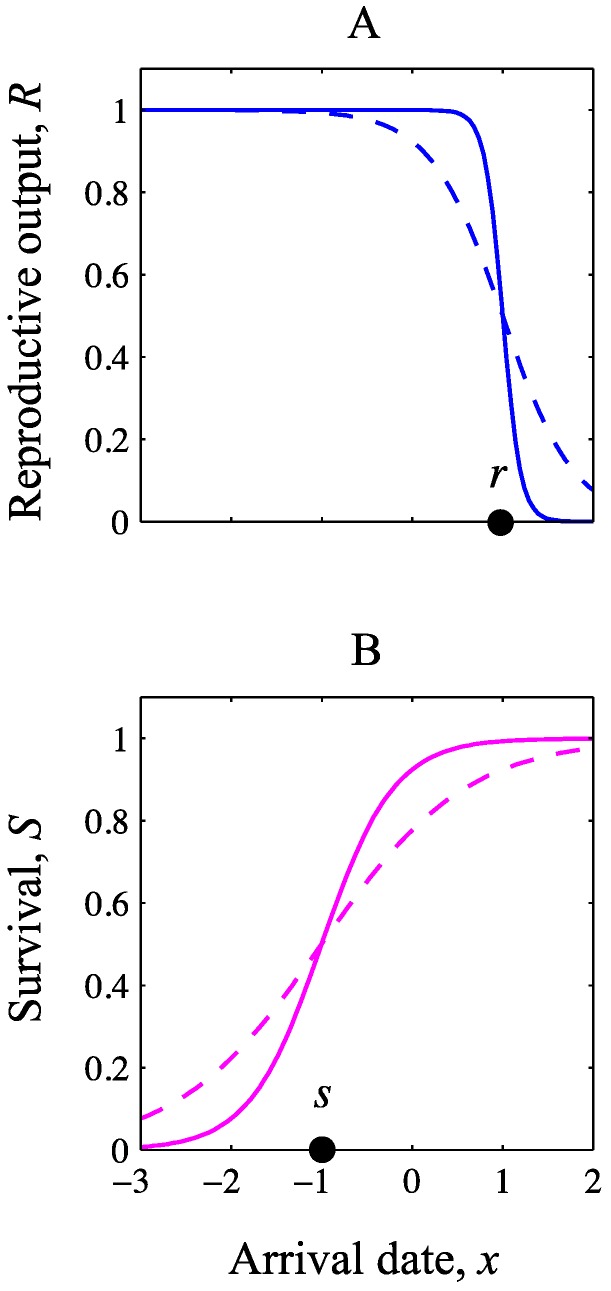
Reproductive output and survival as functions of arrival date. Reproductive output *R* is assumed to decrease with arrival date since late arrival implies late breeding in relation to the food peak date. (**A**) A narrow food abundance distribution gives a sudden reduction (A, solid, blue line) in reproductive output whereas a broad food abundance gives a more gradual reduction (A, dashed, blue line). Survival *S* increases with arrival date due to improved environmental conditions (**B**). The half saturation points, interpreted as food peak date (*r*) and onset of spring (*s*), respectively, are marked with solid circles. The rate of improvement may be fast (B, solid, pink line) or slow (B, dashed, pink line). Parameters: *s* = −1, *r* = 1, *S*_0_ = 1, *R*_0_ = 1; for solid lines *σ_R_* = 0.1 and *σ_S_* = 0.4; for dashed lines *σ_R_* = 0.4 and *σ_S_* = 0.8.

We consider a population of long-distance migratory birds that compete for territories at their breeding grounds. Arrival date at the breeding grounds of an individual is denoted *x*. The beginning and the end of the season are denoted *x_b_* and *x_e_*, respectively. We assume birds raise their young on a temporally restricted resource, such as insect larvae, that is available for only a short period, e.g., [23]. Following Johansson and Jonzén [21], the matching between timing of reproduction and the temporal food abundance distribution is not modelled explicitly. Instead we assume that early arriving individuals can access and use these food resources for a longer period of time compared to later arriving individuals, and thereby obtain a higher reproductive output. With a sufficiently early arrival date, we assume individuals have full access to the food resources and with a sufficiently late arrival date we assume reproductive failure due to lack of food resources. In line with these assumptions we model the expected reproductive success *R* of an individual as a decreasing, logistic function of arrival date (Figure 1A, [21]):

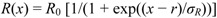
(1)


The parameter *R*_0_ represents the maximal reproductive output. Shifts in the underlying food resource distribution affect the timing of the half saturation point. In this way *r* can be seen as a proxy for the food peak date [21], and to facilitate reading we will refer to *r* as such from this point forward. Finally, small (large) values of *σ_R_* result in steep (shallow) slopes around the inflection point, which we interpret as a narrow (broad) resource distribution (Figure 1A, [21]).

We assume that early migration is associated with mortality costs e.g., due to harsh conditions or low resource levels en route or on the breeding grounds. We let survival *S* be an increasing function of timing of arrival date (Figure 1B, [21]):

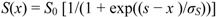
(2)


With this sigmoidal shape we assume that the prospects of survival improve fastest with arrival date around the half saturation point *s*. We assume *s* is associated with (a)biotic conditions such as snow melt or the appearance of food resources and henceforth refer to it as the onset of spring. Note that the survival function derived from first principles by Jonzén *et al*. [20] also has a sigmoidal shape. The parameters *S*_0_ represents the maximal survival rate and the slope factor *σ_S_* controls the rate at which survial increases with arrival date (cf. *σ_R_*). We treat *r* and *s* as separate variables since they represent environmental effects on different parts of the life cycle, which are expected to differ between species [20]. Due to their dependence on shared underlying environmental factors, however, *r* and *s* likely are correlated and we consider both joint and separate changes of these parameters below [21]. 

The population density in a given year *t* is denoted *n_t_*, and is, more specifically, assumed to be measured in terms of migrants returning to the breeding grounds. For simplicity we assume an asexual model. We let *μ* denote the mean arrival date in the population and assume that arrival dates follow the distribution *g_μ_*. We assume that *μ* is under selective control whereas *g_μ_*(*x*) represents spread of arrival dates as a result of environmental variability [24,25]. Following Johansson and Jonzén [21] we study a case where the arrival dates are uniformly distributed. Thus, we assume that *g_μ_* = 1/*v* within the interval [*μ* − *v*/2, *μ* + *v*/2], where *v* is the length of the arrival interval, and 0 otherwise. The main reason to use a uniform distribution is to facilitate the analysis and thereby maximise the transparency of the model (see also [21]). Other distributions of arrival dates, including a Gaussian distribution, have been used to characterize phenology in bird migration data [26]. However, Johansson and Jonzén explored alternatives to a uniform arrival distribution in a similar model (Online Appendix C in [21]) and found that they had only minor effects on shifts of the ESS timing on arrival due to environmental changes. 

Within any given year, the density of individuals that arrives at the breeding grounds increases with arrival date *x*. Density dependence occurs via territory competition among individuals that survive the pre-breeding state. The daily stream of arriving individuals that survive the pre-breeding period equals *g_μ_*(*x*)*S*(*x*)*n_t_*. The accumulated density of surviving individuals, *n_S_*, on a given day is then:

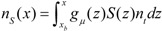
(3)
and the total density of surviving individuals after arrival of all individuals is defined as *N_S,t_* (note that *N_S_*_,*t*_ < *n_t_*) and is given by:

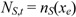
(4)


The majority of the population is assumed to be monomorphic with respect to the mean arrival date. Following the adaptive dynamics framework for evolution of continuous traits [27,28], it is assumed that the prevailing trait is subject to invasions of variant traits due to mutation or potentially migration. It is assumed that invasions are rare, and that the environment is otherwise stable such that population dynamics equilibrize between invasion attempts. The prevailing mean arrival date in the population is denoted *μ* and the variant trait is denoted *μ*'. 

The expected number of offspring *w*(*x*) of a bird with arrival date *x* is assumed to depend on territory quality *Q*(*x*) such that the per capita reproductive output is *R*(*x*)*Q*(*x*). We interpret *R*(*x*) as the reproductive output in the best available territory and interpret *Q* as a discount factor with values between 0 and 1 [21]. We assume that territory quality decreases as individuals occupy the best available territories according to a principle of prior residency [19]. For simplicity we assume that territory quality declines linearly, until 0, with the density of arriving (and surviving) individuals:

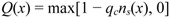
(5)
where the parameter *q_c_* controls how fast the territory quality declines with density. Individuals of the variant population that arrive outside the interval of arrival for the prevailing strategy are assumed to be few and to have a marginal ecological effect, in line with a typical assumptions of the adaptive dynamics approach [22,27,28]. Following Johansson and Jonzén [21] we thus assume *Q*(*x*) = *Q*(*μ − v*/2) for *x* < *μ − v*/2 and *Q*(*x*) = *Q*(*μ + v*/2) for *x* > *μ + v*/2.

The reproductive output in a population with distribution *g_μ_*_'_ is obtained by integrating the product of surviving adults and their reproductive success over the distribution of arrival dates in the population:

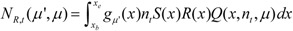
(6)


Population density is reduced by mortality on wintering grounds and during migration. In the model this is captured by the following dynamics between years:

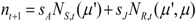
(7)
where the constants *s_A_* and *s_J_* denote winter survival of adults and juveniles respectively. We assume however that changes in *s_A_* and *s_J_* due to environmental changes are strongly correlated. For simplicity we thus let *s_J_* = *ks_A_* with *k* < 1 and consider changes in *s_A_* only. The combined within-year and between-year population dynamic can finally be written as:

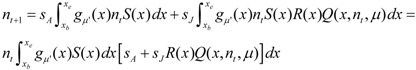
(8)


The age structure in the population is studied by considering the fraction of young in the population, based on equation 7. The first term in equation 7 represents density of adults that have survived migration to the breeding grounds, and the second term represent the density of individuals born in year *t*, *i.e.*, first year migrants that survived the migration to the breeding grounds. Thus we define the fraction of young, *f*, as the fraction of first year migrants within the population of migrating individuals as:

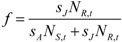
(9)


### 2.2. Analysis

We analyze the model following the adaptive dynamics framework [27,28]. Thus we derive invasion fitness *W*(*μ*',*μ*), understood as the per capita growth rate of the rare invading strategy *μ*' into a population employing strategy *μ*, from equation 8 as:

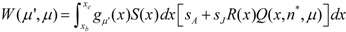
(10)
where n^*^ denotes the population density at equilibrium. This equilibrium was obtained by numerical iterations of equation 8 (with 1,000 time steps). With a uniform distribution *W* can be written:

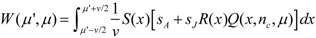
(11)


Following Geritz *et al*. [25] we then calculate the selection gradient *h* as:

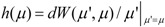
(12)


Assuming that rate of evolutionary change is proportional to *h,* an end point for evolution is then found where the selection gradient vanishes (*D*[*μ*] = 0), provided that this point is also convergence stable and resistant to invasions. Following McGill & Brown [28] we refer to the mean arrival date fulfilling these criteria as an ESS. Note that apart from the assumption of a monomorphic resident population, our analysis is equivalent to analyses based on the quantitative genetic approach to studying gradual evolution under frequency dependent selection [28,29,30]. 

The right hand derivative (indicated by ∂^+^ and defined for *μ*' > *μ*) and the left hand derivative (indicated by ∂^−^ and defined for *μ*' < *μ*) of equation 11 with respect to the variant trait are:

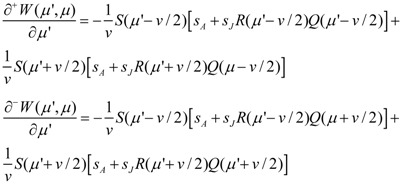
(13)


Since these derivatives coincide when *μ*' = *μ* the selection gradient can be written as [cf. 21]:

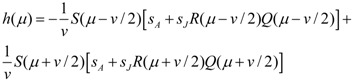
(14)


We found the ESS values by using numerical calculations to solve *h*(*μ*) = 0 and to establish that these points were resistant to invasions and convergence stable [27]. The corresponding equilibrium densities and equilibrium population structures were also calculated numerically.

### 2.3. Ecological and Evolutionary Equilibrium

**Figure 2:**
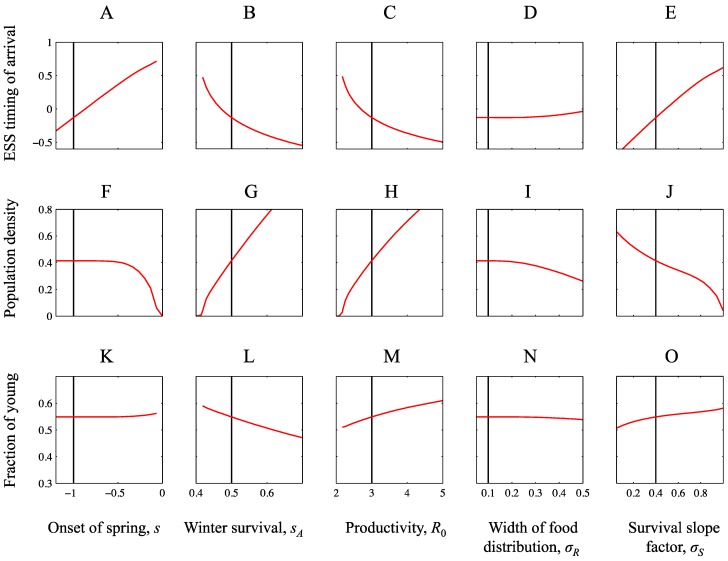
Properties of the ecological and evolutionary equilibrium. The panels show how ESS timing of arrival (**A**–**D**) and the corresponding equilibrium population density (**E**–**H**) and fraction of young at equilibrium (**I**–**L**) depend on model parameters. When not varied as indicated by the graphs, the parameters under study are set to *s* = −1, *s_A_* = 0.5, *R*_0_ = 3 and *σ_R_* = 0.1. These values are indicated by the vertical line which thus represents the same parameter settings across panels. Remaining parameter values: *r* = 1, *S*_0_ = 1, *q_c_* = 1, *v* = 0.5, *σ_S_* = 0.4, *k* = 0.5 (note that *s_J_* = *ks_A_*).

We assume that the population is in a state of ecological and evolutionary (ESS) equilibrium before the environmental change takes place. This equilibrium can thus be interpreted as an historical baseline [22]. The purpose of this section is to characterise how this historical baseline depends on model parameters, and thereby to provide the necessary background for understanding the effects of environmental changes described in the result section. The ESS mean arrival time predicted by the model depends on different environmental parameters in broad agreement with previous models [20,21,22]. Timing of arrival is early when spring starts early since that allows the territory game to take place early in the season (Figure 2A). Winter survival and productivity on the breeding grounds increase competition which in turn favours early arrival (Figure 2B,C). Broadening the resource peak (increasing *σ_R_*) causes the ESS timing of arrival to be slightly later (Figure 2D). This pattern can be due to better reproductive conditions for late arriving individuals, as explained by Jonzén *et al*. [20]. With the parameter settings here, however, individuals arrive before *r* (mean timing of arrival is around 0 and *r* = 1 in Figure 2D), and thus a broader resource peak means fewer resources for reproduction and a lower population size (Figure 1A, Figure 2I). Hence, here the later ESS timing of arrival is due to reduced competition. With a slower improvement of pre-breeding survival (increased *σ_S_*) we also get a later ESS (Figure 2D). This is also an effect of lower competition, due to reduced survival and thereby lower population densities (Figure 2J), since mean timing of arrival occurs after the inflection point *s* (note that *s* = −1 in Figure 2E, cf. Figure 1B). It should be noted that the shape of the pre-breeding survival curve can affect the relationship between the ESS arrival time and the width of the food distribution, which may have a maximum or a minimum (see [20] for details). 

Equilibrium population densities and equilibrium age structure in ESS situations follow in a straightforward manner from the underlying biology. Population density increases with earlier spring (Figure 2F), since survival *S* increases, at least slightly, for any given arrival date. Population density also increases with winter survival (Figure 2G) and with productivity (Figure 2H). Population density decreases with width of the food distribution (Figure 2I) and with slower rate of improvement of pre-breeding survival (Figure 2J), since individuals arrive before *r* and after *s*, as mentioned above. Age structure at ESS is only marginally affected by earlier onset of spring (Figure 2K). This may seem a bit surprising since, at least in the short run, earlier spring favours adult survival, and therefore can be expected to change the age structure. The ESS timing of arrival occurs earlier in the year, however (Figure 1A), which in turn counteracts that effect. Age structure is affected by winter survival and productivity on the other hand. When winter survival increases (Figure 2L) the proportion of adults increases which causes the fraction of young to decrease. In contrast, when productivity increases, the fraction of young increases (Figure 2M) due to an increase in per capita reproduction so that more young are recruited into the population. Broadening the resource peak causes a (weak) decline in fraction of young (Figure 2N), due to the reduction in reproductive success as described above. With a slower rate of improvement of pre-breeding survival the fraction of young decreases however (Figure 2O), due to the reduced survival of adults as described above.

### 2.4. Studying Effects of Environmental Change in This System

We study the effects of change of the environmental variables *s* and *r*. In general, environmental variables may undergo sudden shifts, gradual changes and stochastic fluctuations [31,32]. For simplicity we here consider sudden shifts only, but by envisioning gradual or stochastic changes as a series of smaller shifts their qualitative effects can still be discerned from the results.

When the environment changes, the population will no longer be at ecological and evolutionary equilibrium. In the short term, the population densities may increase or decrease, as may the fraction of young in the population. We record these two properties, along with the new selection gradient *h*(*μ*) after the population density has settled on the new equilibrium. We assume however that evolutionary change, *i.e.*, change in mean arrival time *μ*, is slow compared to demographic responses, and therefore assume that it is not affected. 

## 3. Results

**Figure 3:**
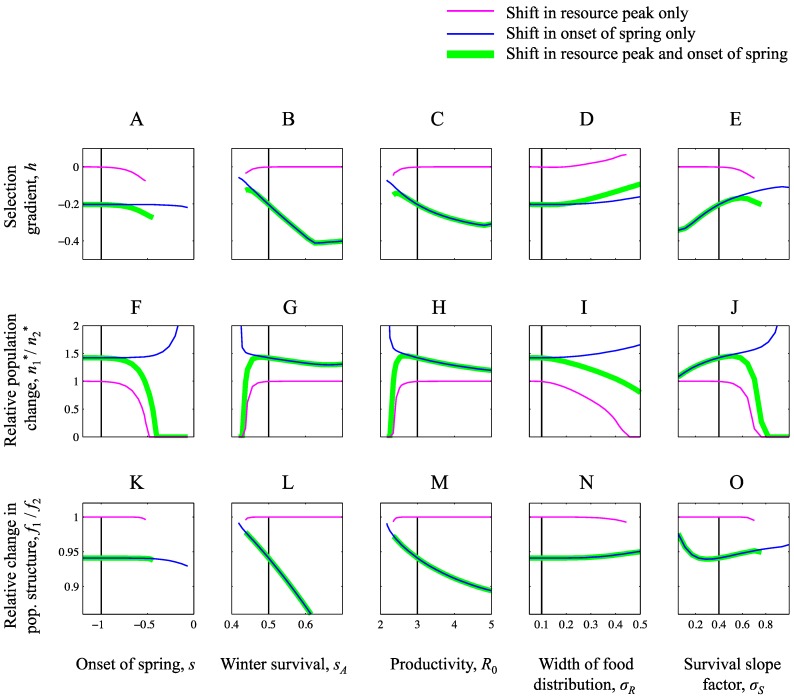
Selective and demographic responses to changed seasonal environments. The figure shows effects of shifts in the timing of the resource peak (*r*, purple), in the onset of spring (*s*, blue) and joint shifts in both these components of the seasonal environment (*s* and *r*, green). The top row of panels (**A**–**E**) shows how selection gradients (from Equation 12) changes due to these environmental changes. Note that the selection gradient is equal to zero for the ESS prior to change. The middle row of panels (**F**–**J**) shows the relative population change, defined as *n*_2_^*^/*n*_1_^*^ where *n*_1_^*^ and *n*_2_^*^ denote the equilibrium density before and after the change, respectively. The bottom row of panels (**K**–**O**) shows the relative change in population structure, defined as *f*_2_/*f*_1_ where *f*_1_ and *f*_2_ is the fraction of young at equilibrium before and after change, respectively. Absence of effect corresponds to selection gradients equal to zero, and the relative changes in population density and population structure equal to one. In cases where population density is zero after the change, the lines in corresponding panels are discontinued. The parameters *s* and *r* were reduced with 0.5 units in the scenarios in which they changed. Parameter values are otherwise as in Figure 2, and with the vertical solid line indicating the same parameter values across panels.

We here account for and compare the effects of temporal advancement of (i) the food peak *r* only, (ii) the onset of spring *s* only and (iii) the food peak *r* together with the onset of spring *s* (Figure 2). We consider, in turn, effects on selection pressures (Figure 3A–E), effects on population densities (Figure 3F–J) and effects on population structure (Figure 3K–O). Each of these effects are studied for the range of populations with different historical baselines shown in Figure 2. Each panel in Figure 3 shows the effects of environmental changes on the property in the corresponding panel in Figure 2. The relative sensitivity of the biological properties to the different types of environmental changes (i–iii) can be discerned by comparing the different effects (purple, blue and green lines in Figure 2).

### 3.1. Ensuing Selection Pressures after Seasonal Changes

An advanced onset of spring has a strong effect on selection compared to an advanced food peak when spring is already early (Figure 3A), when winter survival is high (Figure 3B), when productivity is high (Figure 3C), when the resource peak is narrow (Figure 3D) and with slow improvement of pre-breeding conditions (Figure 3E). In these cases, the selection after only *r* has changed is very small (solid lines are close to zero), and the effect of changing *s* and *r* jointly (green lines) is very similar to changing *s* only (blue lines).

Under most settings, advanced food peak leads to selection for earlier arrival (Figure 3A–C,E). When the resource distribution is broad, however, advanced food peak can cause selection for a delayed arrival date (Figure 3D). To understand this phenomenon, note that a changed resource peak affects the adaptive landscape not only by affecting the reproductive success of individuals for different arrival dates, but also by changing the reproductive success as such (Figure 1A), which, via effects on population densities, can affect the competitive pressure. At evolutionary equilibrium there is a balance between selection for earlier arrival to increase reproduction and to avoid competition, and selection for later arrival dates to increase survival (cf. Equation 14). When the food peak advances, selection for earlier reproduction increases but population density decreases (Figure 3I). Thereby also the competitive pressure decreases, which in turn weakens selection towards earlier arrival dates. The effect of a food peak shift on selection is relatively weak when the food distribution is wide, but the effect of population densities (Figure 3I) and thereby on competition may still be substantial, which explains why later arrival can be favoured under these circumstances.

Finally we note that *s* has the stronger influence on selection when the ESS is early (compare Figure 3A–E with Figure 2A–E). An early ESS can in turn occur either when spring is early or when competition is strong. An early spring leads to a large population, whereas strong competition occurs when the population density is large e.g., due to high winter survival, high productivity, narrow resource distribution or slow rate of improving pre-breeding conditions. Thus, strong influence of *s* on selection is also predicted by large population size (Figure 2F–J).

### 3.2. Equilibrium Population Density after Seasonal Changes

The population grows when spring advances and declines when the food resource peak advances in all scenarios (Figure 3F–J). The effect of changed resource peak is very small for many parameter settings investigated (purple lines are close to 1 in large parts of Figure 3F–J), however. In these cases, the model predicts a net increase when both factors advance, indicating that the positive effects on population density from the advanced spring overrides the small negative effects of an advanced food peak. More precisely, this occurs when the baseline ESS mean arrival time is early, which, as discussed above, is in turn predicted by large population densities. In cases where changes in *r* have the larger effect, namely at low population densities in the baseline (cf. Figure 2F–J), the changes in *r* can be very strong however, and lead to strong reductions or even extinctions of the population (Figure 3F–J).

### 3.3. Equilibrium Population Structure after Seasonal Changes

The fraction of young in the population decreases both when the onset of spring advances and when the food peak advances. In many cases, the effect of changed resource peak is again very small, however (purple lines are close to 1 in large parts of Figure 3K–O). Effects of *s* and *r* on population structure follow the same pattern as the effects of *s* and *r* on selection and population density: high population densities (Figure 2F–J) predict more effect of *s* than of *r*.

## 4. Discussion

### 4.1. Summary of the Results and Their Significance

Our eco-evolutionary approach to studying phenological adaptation in migratory birds contributes with several new insights into how demographic changes relate to phenological shifts in migratory birds. To start with, our analysis allows for a comparison of the effects of advanced onset of spring (decreased *s*) and advanced food peak (decreased *r*) on total population density. Since advanced onset of spring alone increases pre-breeding survival and advanced food peak reduces reproductive success, the two types of environmental change have opposing effects on population density. When both changes occur in parallel, e.g., reflecting even temperature changes over the season, the net population trend can thus be hard to predict. The model shows, however, that the historical baseline influences which of the two factors has the largest influence. Thus, when the ESS timing of arrival time is early, e.g., due to strong territory competition, the positive effects of improved pre-breeding conditions are strong and we get a net population increase. When the ESS arrival time is late, the negative effects of reduced reproduction are stronger and we get a net population decline.

In many cases, our model predicts weak effects of a changed resource peak on population trends (Figure 3F–J). This observation seems to go counter the emphasis on changed resource peaks to explain population trends in migratory birds [6]. On the other hand, we can also see from our results that, whereas the positive effects of an advanced onset of spring in our model only increases population densities a little, a changed resource peak can cause strong decreases in population size and even extinctions. In this way, our model support concerns that mismatched reproduction can indeed have serious consequences for populations of bird species. Our model moreover suggests indicators for situations where shifts in the food distribution are expected to have strong negative effects on populations, e.g., low winter survival (Figure 3G), low productivity (Figure 3H), and slow improvement of pre-breeding conditions (Figure 3J).

Another interesting result is that the fraction of young decreases both when the resource peak advances and when the onset of spring advances. This result is not surprising when one considers the fact that this measure does not inform on the absolute numbers of young: increased reproductive mismatch (decreased *r*) reduces the number of young recruited into the population and improved survival due to earlier onset of spring (decreased *s*) increases the proportion of adults. On the other hand, this result shows that relating phenological shifts solely to food resource peaks can be misleading when interpreting associated demographic responses [9]. Under the assumption that phenological shifts mostly affect demography via reproduction, a reduced fraction of young in a population would be interpreted as a sign of trophic mismatch, whereas in the light of our study, it could well be due to improved pre-breeding conditions. In many cases, our model also predicts a relatively small effect of an advancing food peak on population structure (Figure 3K–O), which reduces the scope for explaining changed population structure with shifts in food resource peaks even further. 

When it comes to evolutionary responses to climate change, our analysis highlights the role of pre-breeding survival for phenological adaptation in migratory birds. As it turns out, the survival costs not only modulate responses to changed food peaks, as shown by Jonzén *et al*. [20], but also act as a key factor for selection on arrival dates on its own. In many cases there is almost no selective response to changes in the resource peak (*r*), but there are always selective responses when the onset of spring (*s*) changes (Figure 3A–E). According to our model, adaptive changes in arrival times are more likely to be associated with changes in pre-breeding conditions rather than with changes in food peaks. 

In the predecessors [20,21,22] of the model developed here, competitive pressure was treated as a parameter. In the present model, competitive pressure instead depends on the underlying ecology in the model and the strategies employed by the population. We have thereby closed the eco-evolutionary feedback loop. A first insight from this more complete treatment is that parameters that affect the equilibrium population density, such as winter survival and productivity, by adjusting the competitive pressure, also affect the evolutionarily stable timing of arrival (Figure 3B,C). We have also shown that advancing the resource peak can favour later arrival dates under the influence of eco-evolutionary feedbacks (Figure 3D). This example of selection counter the direction of environmental change underscores the fact that competitive interaction can affect phenological adaptation in non-intuitive ways and contributes a new adaptive mechanism to explain variation in phenological trends.

The enhanced realism in the model analysed here also allows a closer connection to empirical observations, compared to previous models of this kind. Below we compare our model predictions with phenological responses in the Pied flycatcher. Thereafter we discuss different possibilities to further explore this eco-evolutionary approach to studying phenological adaptation in a changing climate.

### 4.2. Comparisons with Observed Patterns of Phenological Adaptation

A general result from our study is that ecological differences can cause variation in adaptive phenological responses to climate change. Naturally, species-specific properties will give rise to a certain amount of variation in phenology. Therefore, to reduce confounding factors, our hypotheses should be compared to phenological responses within a single species. Interestingly, breeding populations of pied flycatchers, *Ficedula hypoleuca*, have shown markedly different responses to environmental change across Western Europe [33]. The pied flycatcher is a long-distance migrant that breeds in Eurasia in summer and overwinters in Africa, south of the Sahara desert [34,35]. In The Netherlands, population numbers have declined in areas where a large mismatch exists between the timing of egg-laying and peak in food availability [36]. The timing of arrival did not change but the laying date has advanced. However, this change has been insufficient to avoid demographic consequences, as shown by declined population numbers [5]. Pied flycatchers in Britain also show large declines in population numbers. A study by Goodenough *et al*. [12] suggested, however, that only part of this decline can be explained by reduced breeding performance. 

In a study on Pied flycatchers in Finland it was found that laying date was delayed in comparison to measures of spring phenology [37]. Judging from these trends, and the fact that clutch sizes also decreased, Laaksonen *et al*. [37] speculate that some trophic mismatch has occurred. The density of breeding pairs showed a quadratic trend over the study period, however, with a minimum for intermediate years [38]. Thus, the increased mismatch has been accompanied by an increase of the population towards the end of the study period.

A first observation we can do from these studies is that the mismatch between laying date and resource food peak alone does not predict trends in population change. This is in line with a general result of our study that the effect of changed food peak date on population densities may vary significantly depending on the historical baseline and on changes in other environmental variables (Figure 2).

Some of the differences between these responses can also be related to factors that influence demographic responses in our model. For the British population, for example, there is a relatively long time interval between arrival and breeding (around 30 days [39]). In relation to our model, this could be interpreted as an historical adaptation to a relative large difference between *s* and *r*. Under these circumstances, our model predicts a relatively small effect of a changed resource peak on population density (low value of *s* in Figure 3F). In the Dutch population in contrast, there is a short time interval between arrival and breeding (around 10 days [5]). This is a situation where the model predicts a relatively strong effect (high value of *s* in Figure 3F). In this way, the model predictions are in agreement with population decline, depending little on resource peak changes in Britain and depending more on resource peak changes in The Netherlands, as suggested by these studies. 

It is also interesting to note that one factor that explained the decline in the British population [12] was Winter NAO index, which, as suggested by the authors, is likely to influence food availability on the wintering grounds and during migration. This could be interpreted as reduced winter survival or reduced productivity, which in our model also can explain reduced population density (Figure 2G,H).

For early arriving individuals in the Finnish population, reproductive mismatch and increased population density seems to have occurred simultaneously. This may seem strange, but interestingly, temperatures during the early part of their migration have increased as well [39], which could be interpreted as reduced survival cost for early arrival, *i.e.*, earlier *s* in our model. If the majority of this population arrives early, then this change can indeed explain increased population density in our model (Figure 3F–J), even when trophic mismatch via advanced food peak occurs simultaneously (advanced *r*). In this population, there was also a change in the arrival date for early migrants [39], which is adaptive according to our model when *s* decreases (Figure 3A) and is in line with long term increase of population density as well (Figure 2F).

In sum, we find that a number of observations from these systems are in line with predictions from the model. These comparisons also serve as an illustration for the motivation of our study; namely the need to take the whole life history and a broad set of factors into account when predicting demographic changes involved in phenological shifts in migratory birds.

## 5. Future Research on Eco-Evolutionary Dynamics in Phenological Shifts

We conclude by highlighting some interesting avenues to continue studying phenological adaptation based on the eco-evolutionary approach presented here. While we above make some qualitative comparisons between the model and observed responses, logical next steps are to test the model predictions against a standardized data set for pied flycatchers in Europe and to parameterize the model using detailed data from a well studied population, as in e.g., *Parus* spp. populations in the Netherlands [5,36] and in Britain [40]. Most of the existing studies estimate changes in the food peak (*i.e.*, estimations of *R*) and it would be interesting to include estimations of changes in pre-breeding conditions (*i.e.*, to estimations of *S*) which has been done to a lesser extent (but see e.g., [39]).

There is also much scope for further enhancing the ecological realism of the model. One could for example include features from related annual routine models [16,17,18], such as different stop over sites and more detailed physiology, into the framework. That would enable a more explicit modelling of the migration ecology and could shed light on how other factors than strong survival costs constrain evolution of early arrival. Many *Parus* species and pied flycatchers compete for food and nestboxes [41,42] and this may affect phenological responses. It would therefore be interesting to include interspecific competition into the model. One shall also note that our model does not include breeding time as such. Our assumption of reproduction being a sigmoid function of arrival date means that only arrival in relation to the food resource peak determines reproductive success. In nature, breeding may be hampered by other factors than arrival time however, e.g., inappropriate cues [43]. In other words, whereas suboptimal arrival necessarily confers suboptimal reproduction, optimal arrival does not guarantee optimal breeding. To address this question one could include breeding time as an additional trait into the model. Costs of early breeding relative to the arrival time, e.g., reduced resources or physiological costs for the female [23], could then be represented in the model by reduced reproduction or survival.

In our analysis we studied the ESS timing of arrival and changes in selection pressures following environmental changes. It is however possible to adapt the framework to create predictions on subsequent gradual evolutionary trajectories as well. One may then for example use the canonical equation of adaptive dynamics for mutation limited evolution [44], or relax the assumption of a monomorphic resident population and include genetic variation, following a quantitative genetic approach [45] or individual-based simulations [31]. Note also the fitness function employed here by definition describe an adaptive landscape that can be studied to predict the likelihood of invasions of different traits following environmental changes.

Theories for the evolution of continuous traits under the influence of ecological feedback have developed rapidly over the last decades [28,29,30,46]. It has been argued that they are only just beginning to tap into their potential impact on ecology and evolution, and developing new and better applications is therefore the most important future direction [28]. By developing a framework for studying demographic responses and phenological shifts in a changing climate we have not only taken a step in that direction, but also generalized and extended the theory for adaptation to changed seasonal environments.
